# Hemorrhagic Malarial Fever

**Published:** 1883-08-20

**Authors:** E. H. M. Parham

**Affiliations:** Arkansas


### Southern Medical Record:

EDITORS:

T. S. POWELL, M.D. W. T. GOLDSMITH, M.D. R. C. WORD, M.D.

*R. C. WORD, M.D., Managing Editor.*

☞All Communications and Letters on Business connected with the RECORD must be addressed to the Managing Editor.

VOL. XIII. ATLANTA, GA., AUGUST 20, 1883. No. 8.

### ORIGINAL AND SELECTED ARTICLES.

### HEMORRHAGIC MALARIAL FEVER.

BY E. H. M. PARHAM, M. D., OF ARKANSAS.

In the February number of the SOUTHERN MEDICAL RECORD, page 65, I saw the treatment of hemorrhagic malarial fever, reported by Dr. Joiner, of Andersonville, Ga., taken from the Southern Practitioner, in which he recommends hot water as one of the leading therapeutic agents in the treatment of that dreadful disease. I have not tried it, but believe that it might be attended with some advantage, as the skin and kidneys are the chief organs through which we may expect to relieve the system from the malarial poison already existing; but why the learned Dr. should entertain fears of the free use of calomel, as expressed in that article, I cannot see, as it is well known to be the most potent remedy we have to correct morbid secretion; one of the chief factors in the development of that disease, yet he used one grain per diem, and well he did; as I have not the least doubt but that the calomel and hot water prevented fatal consequences from the free use of quinine. He also said that he had lost confidence in quinine. Yet he ordered 40 grains to prevent an expected chill that evening. I will here remark, from abundant experience, that had the Dr. used the calomel every 4 hours, and omitted quinine altogether, that he would have had the pleasure of seeing his patient convalescent in a much shorter time.

It will be remembered by the readers of the SOUTHERN MEDICAL RECORD, that I reported a case of hemorrhagic fever in 1881, published in the February number of that year. Since that time I have treated many cases, without losing a single one. My special and distinguished friend, Dr. J. K. Hodge, of Princeton, Ark., informed me a short time since, that he had never lost but one case since he adopted the plan of treatment set forth in that article; he first suggested the tincture of the muriate of iron, when ergot could not be used. Dr. Hodge saw the case he lost too late to expect any benefit from medicine, as the case was neglected, and did not live 24 hours from the time of his first visit. I know that Dr. Hodge has treated many cases, and has published his views at length in the Medical Brief, published in St. Louis, Mo., which I fully endorse, although I have not seen it. I obtained the principles set forth in that article from him personally.

It seems to me that it will be well here to give some of the most prominent symptoms by which the above-named disease may be known: It usually appears in subjects that have had repeated attacks of chills, and who have taken quinine alone to break up the paroxysms without paying any attention to the secretions, until constant lassitude with indisposition, and in fact, inability to exercise; the skin now begins to assume a jaundiced appearance, and suddenly the subject is taken with severe rigors, succeeded by fever with a depressed circulation, constant sick stomach, and frequent vomiting; the jaundiced appearance of the skin now increases rapidly in intensity, and thirst is indomitable, and often pure blood discharged every two or three hours from the kidneys. I recollect one case last fall that constant delirium, in addition to the above symptoms, attended, which case made a good and speedy recovery under the treatment which I intend to repeat in this article. Let it be remembered that this is a continued fever and will be speedily fatal if not controlled; I have never seen a case that there was any intermission, therefore I claim that quinine is not indicated, except to neutralize the poison already in the system, which it utterly failed to accomplish. I have repeatedly known the administration of 5 to 10 grains to be followed in an hour with severe rigors, and stop the administration of quinine and we would have no more rigors. The indications to be met are clearly: 1st. To arrest the hemorrhage; 2d. Correct morbid secretion; 3d. Remove renal congestion; 4th, to restore the secretion of bile to its proper channel; and, 5th, also to eliminate from the blood the effete substances already contained therein; the nausea will take care of itself as soon as the cause is removed, and by meeting the above indications the removal of that cause will quietly and surely be accomplished. To meet the 1st indication I give fluid extract of ergot or tincture of the muriate of iron every 4 hours in 10 drop doses, to be given with some diaretic, (I prefer the infusion of buchu leaves) which will meet the 3d indication; and for the 2d and 4th I give one grain of calomel with 3 or 4 grains of Dover's powder every 4 hours, to be given alternately with the ergot or tincture of iron; to meet the 5th and last indication, I give as above directed the infusion of buchu leaves to promote the action of the kidneys and Dover's powder as a diaphoretic.

In one aggravated case last fall, I gave a few doses of tannic acid, I thought perhaps, with some benefit; at least the patient made a good and speedy recovery. This case had profuse hemorrhage from the stomach, bowels and kidneys; I give calomel until the liver begins to throw off bile and symptoms improve. I seldom find it necessary to salivate; I always blister over the liver and stomach, in severe cases, and I think with advantage. Laxatives should be used with caution, as the bowels, when started, are disposed to act too much, and this disease is always attended with great prostration. I prefer mild enemata when an action can be obtained by them.

Absolute quietude should be observed in every case, and the stomach should not be disturbed with any medicine, unless clearly indicated; as those named above. I allow such light and easily digestible articles of diet as the patient's appetite, in each case, seems to call for.

I have written this article hastily, without regard to logic or a desire for notoriety. I am too old for that; but am actuated only by philanthropy, and believe that I am justified in saying that if this is given a thorough trial that it will be universally adopted by the medical fraternity, and that it will save annually the lives of hundreds and perhaps thousands of the good citizens of this country. It might be said that I am speaking extravagantly, but I know that it is no more than the importance of the case demands. I hope that you will find space in your very valuable Journal for its publication.

---

**Flatulence.**—In flatulence, Dr. Bruen [Phila. Hosp.] prescribes a pill containing five grains of bicarbonate of soda and five drops of oil of eucalyptus two hours after meals. Pepsin or pancreatin with milk food, and the mineral acids with meats, should be directed to be taken immediately after meals.—*South. Clinic.*

